# Peritoneal Nebulization of Ropivacaine during Laparoscopic Cholecystectomy: Dose Finding and Pharmacokinetic Study

**DOI:** 10.1155/2017/4260702

**Published:** 2017-02-20

**Authors:** Massimo Allegri, Martina Ornaghi, Catherine E. Ferland, Dario Bugada, Yash Meghani, Serena Calcinati, Manuela De Gregori, Federica Lovisari, Krishnaprabha Radhakrishnan, Maria Cusato, Stefano Scalia Catenacci, Marta Somaini, Guido Fanelli, Pablo Ingelmo

**Affiliations:** ^1^Department of Anaesthesia, ICU and Pain Therapy, University Hospital of Parma, Parma, Italy; ^2^Department of Surgical Sciences, University of Parma, Parma, Italy; ^3^SIMPAR Group (Study in Multidisciplinary Pain Research), Parma, Italy; ^4^Department of Anaesthesia and Intensive Care, San Gerardo Hospital of Monza, Milan Bicocca University, Milan, Italy; ^5^Department of Anaesthesia, Montreal Children's Hospital, McGill University, Montréal, QC, Canada; ^6^Alan Edwards Centre for Research on Pain, Montreal, QC, Canada; ^7^YAP Group (Young Against Pain), Firenze, Italy; ^8^Pain Therapy Service, IRCCS Fondazione Policlinico San Matteo, Pavia, Italy; ^9^Laboratory of Clinical Pharmacokinetics, IRCCS Fondazione Policlinico San Matteo, Pavia, Italy; ^10^Department of Anaesthesia and Intensive Care I, ASST Grande Ospedale Metropolitano Niguarda, University of Milano-Bicocca, Milan, Italy

## Abstract

*Background*. Intraperitoneal nebulization of ropivacaine reduces postoperative pain and morphine consumption after laparoscopic surgery. The aim of this multicenter double-blind randomized controlled trial was to assess the efficacy of different doses and dose-related absorption of ropivacaine when nebulized in the peritoneal cavity during laparoscopic cholecystectomy.* Methods*. Patients were randomized to receive 50, 100, or 150 mg of ropivacaine 1% by peritoneal nebulization through a nebulizer. Morphine consumption, pain intensity in the abdomen, wound and shoulder, time to unassisted ambulation, discharge time, and adverse effects were collected during the first 48 hours after surgery. The pharmacokinetics of ropivacaine was evaluated using high performance liquid chromatography.* Results*. Nebulization of 50 mg of ropivacaine had the same effect of 100 or 150 mg in terms of postoperative morphine consumption, shoulder pain, postoperative nausea and vomiting, activity resumption, and hospital discharge timing (>0.05). Plasma concentrations did not reach toxic levels in any patient, and no significant differences were observed between groups (*P* > 0.05).* Conclusions*. There is no enhancement in analgesic efficacy with higher doses of nebulized ropivacaine during laparoscopic cholecystectomy. When administered with a microvibration-based aerosol humidification system, the pharmacokinetics of ropivacaine is constant and maintains an adequate safety profile for each dosage tested.

## 1. Introduction

Postoperative pain remains a major problem after laparoscopy, having direct consequences on the patient's quality of life as well as increasing health care costs [1, 2]. Pain after laparoscopic surgery is usually attributed to surgical manipulations, including intraperitoneal insufflation of carbon dioxide, resulting in peritoneal stretching, diaphragmatic irritation, changes in intra-abdominal pH, and retention of the insufflated gas within the abdominal cavity after surgery [3]. These manipulations also result in the irritation of peritoneal nerves causing visceral and shoulder pain. Previous studies have reported that as many as 80% of patients required opioid analgesia after laparoscopic surgery [1, 4, 5], which also may increase postoperative morbidity and hospital stay [2].

Intraperitoneal nebulization of local anesthetics through microvibration-based aerosol humidification devices combines the analgesic benefits of gas conditioning (humidification) and local anesthetic instillation, allowing for a uniform dispersion of the solution throughout the peritoneum [6]. This method has shown great efficacy in the control of postoperative pain [7–12] and in the reduction of postoperative morphine consumption when compared with both placebo and peritoneal instillation in different clinical scenarios [12–14]. However, in all those studies there were patients experiencing residual pain and requiring significant amount of opioids. Moreover, there was no formal analysis of the least effective dose of local anesthetic.

Pharmacokinetic studies on animal models have shown that intraperitoneal cold nebulization of ropivacaine is a safe technique, and the drug's kinetics is similar to the instillation method [7, 15]. Human studies have confirmed favorable pharmacokinetics and pharmacodynamics of nebulized ropivacaine. Peak concentration is attained between 10 and 30 minutes following the end of aerosolized ropivacaine delivery and the plasma concentration of local anesthetics remained within safe levels even after nebulization of 3 mg/kg of ropivacaine [16]. However, the delivery of high doses of ropivacaine (i.e., 3 mg/kg) may be unnecessary or even impractical due to the long nebulization time with the actual commercially available systems. Moreover, it is possible that lower effective doses could have less and different kinetics due to the postnebulization loses during normal (less controlled) surgical procedures [17].

The aim of this multicenter, double-blind, randomized, controlled phase III clinical trial was to assess if an increase on the doses of nebulized ropivacaine is associated with an increase on its analgesic efficacy expressed through the reduction of morphine consumption after laparoscopic cholecystectomy. As a second aim, we evaluated the possibility of a dose-dependent systemic absorption of ropivacaine, when nebulized in the peritoneal cavity during everyday clinical conditions.

## 2. Methods

After Ethics Committee approval by the two hospitals involved in the study (San Gerardo Hospital, Monza and IRCCS Policlinico S. Matteo, Pavia, Italy), this trial was registered on clinicaltrials.gov (NCT01143025 06/09/2010). The study was designed according to the CONSORT guidelines (https://www.consort-statement.org).

### 2.1. Patients

All patients in this study provided written informed consent. Eligible patients were adults between 18 and 75 years of age, had an American Society of Anesthesiologist physical status score of I–III, and were scheduled for elective laparoscopic cholecystectomy. Patients were excluded if they had a clinical diagnosis of acute pancreatitis, acute preoperative pain other than biliary colic, chronic pain treatments, or antiepileptic treatment, history of alcohol or drug addiction, severe hepatic or renal impairment, allergy to the study drugs, or cognitive impairment or communication problems or were pregnant or lactating.

As the goal of this trial was to evaluate the effect of intraperitoneal ropivacaine nebulization, conversion to an open technique was considered a protocol dropout. Dropout patients were excluded from the efficacy analysis but received the same analgesia protocols and evaluations until their hospital discharge for the safety analysis.

### 2.2. Randomization Procedures

Patients were randomized with a computer-generated randomization sequence into three groups: peritoneal nebulization of 5 mL (50 mg group), 10 mL (100 mg group), or 15 mL (150 mg group) of ropivacaine 1%. The coordinators of the research centres received a package containing sequentially numbered, opaque, sealed, and stapled envelopes during the research team meetings. The anesthesia nurse received the sealed envelope containing allocation number and instructions for the solution preparation from a research assistant before patients entered the operating room. Corresponding envelopes were opened only after the enrolled participants completed the identification checklist. The research assistants involved in data collection and the anesthesiologist and the surgeon of the case (not involved with the study) were unaware of the study group assignment. The blinding was disclosed only after the statistical analysis. In case of an emergency related or possible related to study drugs, the nurse or the research assistant of the coordinator centre was authorized to disclose the content of the syringe to the anesthesiologist and clinicians. The research assistants involved in data collection as well as nurses and doctors who had direct patient contact were unaware of the study group assignment.

### 2.3. Anesthesia Procedures

A standard anesthetic technique was used for all patients. Patients were premedicated with diazepam 5–7 mg, 30 minutes before surgery. General anesthesia was induced with propofol 2-3 mg kg^−1^ IV and tracheal intubation was facilitated with cisatracurium 0.15 mg kg^−1^ IV. Anesthesia was maintained with sevoflurane 1.5–2.5% end-tidal concentration titrated to maintain state entropy values between 45 and 60 (Entropy sensor™, M-ENTROPYTM module, GE Healthcare, Helsinki, Finland), fentanyl boluses 1-2 mcg kg^−1^ titrated to maintain noninvasive mean arterial blood pressure and heart rate ±20% of basal values, and cisatracurium 0.03 mg kg^−1^ titrated to maintain a train of four count (TOF) of 1-2, as well as according to clinical needs. Ventilation was controlled to maintain end-tidal CO_2_ between 35 and 40 mmHg. After tracheal intubation, an orogastric tube and an oesophageal temperature probe were placed. The operating room temperature was set to 20°C, and patients were kept warm using a forced warm-air device and warmed intravenous solutions. At the end of surgery, residual muscle paralysis was reversed with neostigmine 0.05 mg kg^−1^ and atropine 0.02 mg kg^−1^, and tracheal extubation was performed once clinical signs were observed and a TOF ratio of 0.9 was achieved.

Surgery was performed with the 4-access technique; the pneumoperitoneum was obtained through nonheated, nonhumidified CO_2_. Nebulization was performed from the beginning of the pneumoperitoneum through the main trocar using Aeroneb Pro® nebulizer, while the other ports were being inserted until the end of the available dose. The nebulization unit was placed in series between the insufflator and the insufflation tubing.

All patients received dexamethasone 4 mg IV after the induction of anesthesia and ondansetron 4 mg IV at the end of surgery for postoperative nausea and vomiting prophylaxis. A bolus of paracetamol 15 mg/kg (up to a maximum of 1 g) was administered during surgery together with wound infiltration with 3 mL of ropivacaine 0.5% after completion of surgery.

### 2.4. Analgesia Protocol

Upon arrival to the postanesthesia care unit (PACU) morphine 3 mg boluses were administered in order to obtain a numerical rating score (NRS) less than 3 points on a 0–10 scale, of which 0 represented “no pain” and 10 represented “worst possible pain.” In the surgical ward analgesia was provided with IV paracetamol (1 g every 6 hours) and patient controlled analgesia (PCA) with morphine (1 mg bolus, 5-minute lock-out, and 4 hours of maximum dose of 20 mg) during the first 48 hours. This allowed us to measure morphine consumption more accurately. Ondansetron 4 mg was administered every 12 hours for PONV prevention. Patients were encouraged to ambulate as soon as possible. On the basis of our routine practice, all patients remained in the hospital for 48–72 hours.

The primary endpoint of this study was the total consumption of morphine (PACU and ward) during the first 48 hours after surgery. We also assessed the proportion of the patients requiring morphine during the first 24 hours and 48 hours after surgery.

Collected data included patient age, gender, weight, and height, intraoperative opioid use, duration of surgery, postnebulization volume of ropivacaine (residual volume in the nebulization unit plus the volume in the silicon connection tubes), signs of local anesthetic toxicity (e.g., intraoperative arrhythmias, unexplained hypotension, burst suppression on entropy monitor, and unexplained delayed awakening), patient temperature in the PACU, and duration of PACU stay. Patients were evaluated in PACU and at 4, 6, 24, and 48 hours after nebulization. At each control, we registered pain intensity static pain (at rest) and dynamic pain (on deep breathing, coughing, or movement), using the NRS, discriminating between general, abdominal, wound, and shoulder pain. Morphine consumption, time to unassisted ambulation, discharge criteria according to modified postanesthetic discharge scoring system (PADSS), and possible adverse effects such as shivering, nausea, or vomiting were also collected.

### 2.5. Pharmacokinetic Assessment

The analysis of ropivacaine pharmacokinetics was structured as a single dose study. Ropivacaine plasma concentrations were determined on venous blood samples collected before the procedure and 20, 40, 60, 90, 240, and 360 minutes after the end of nebulization. Each sample (10 mL) was collected into a tube containing EDTA and immediately centrifuged at 3000 rpm for 10 minutes. Each plasma sample was then transferred into 2 polypropylene tubes and sealed and stored at −20°C until analysis. All analyses were conducted at the Laboratory of Clinical Pharmacokinetics of the IRCCS Fondazione Policlinico San Matteo, Pavia.

The following parameters were evaluated: total and free ropivacaine plasma concentrations, with estimation of peak concentrations (*C*_max_), time to peak concentrations (*T*_max_), absorption rate constant (*k*_*a*_), absorption half-life (*t*_1/2_ abs), elimination half-life (*t*_1/2_) and elimination rate constant (*k*_*e*_), and area under the concentration-time curve of the drug from the time of dosing to 6 h later (AUC_0–6 h_). Total and free ropivacaine concentrations were assessed using high performance liquid chromatography validated for precision, accuracy, linearity, specificity, and recovery.

### 2.6. Statistical Analysis

This study consisted of a continuous response variable (morphine consumption). In a previous study [18], the response within patients receiving preoperative nebulization was normally distributed with a mean morphine consumption 48 hours after surgery of 12 mg ± 15 mg. Based on Student's two-sample *t*-test, if a true difference in the experimental and control means morphine consumption is 6 mg, therefore a minimum of 50 experimental subjects per group would be needed to reject the null hypothesis that morphine consumption 48 hours after surgery of patients receiving nebulization of ropivacaine 50, 100, and 150 mg is equal, with a power of 0.9 and Type I error = 0.05 (2-sided) (PS Power and Sample Size Calculations© Version 3.0, January 2009). Fifty-five patients were enrolled in each group to allow for protocol violations.

Continuous variables (age, surgery duration, intra-abdominal pressure during pneumoperitoneum, CO_2_ volume, nebulization duration, residual volume, effective nebulization volume, percentage of nonadministered volume, body temperature variation, PACU permanence duration, morphine consumption, intensity of postoperative general pain and pain localized to abdomen wound and shoulder, time of unassisted ambulation, and time of hospital discharge) were presented as means and standard deviations and were analyzed with ANOVA.

Discrete variables (sex, drain presence, morphine use, time of unassisted ambulation, m-PADDS score ≥ 9, presence of adverse events, or other complications) were reported as percentages and were analyzed with either the chi-square or Fisher exact test. Statistical analyses were performed with Epi Info 2007 (Epi Info, CDC, Atlanta, USA).

Pharmacokinetic data were analyzed as group means with standard deviation. We calculated the mean of nebulization time, the average and percentage drug loss, *C*_max_ for each group, the number of patients achieving this value, and the maximum concentration stratified by group. ANOVAs were used to examine the differences in pharmacokinetic parameters of anesthetics. A *P* value < 0.05 was considered statistically significant for all tests. AUC_0–6_ was calculated using the trapezoidal rule from 0 to 6 hours. Pharmacokinetic calculations were performed using the Kinetica 4.0 software (INNAPHASE Corporation, Philadelphia, PA, USA).

## 3. Results

One hundred and seventy-six patients were enrolled during the preoperative evaluation. Eleven patients did not receive the intervention during surgery due to logistical difficulties (mainly lack of nebulization units). Six patients were excluded from the efficacy analysis, one in the 50 mg group, three in the 100 mg group, and two in the 150 mg group. Five of them were converted to open technique; one disclosed a previous history of drug abuse after receiving the intervention. One hundred and fifty-nine patients completed the study and the efficacy analysis. The pharmacokinetic study included 36 patients (12 patients from each group) of the 165 patients recruited from the main study.

There were no clinical or statistical differences between groups in age, sex, duration of surgery, intra-abdominal pressure during pneumoperitoneum, CO_2_ volume, temperature variation, need of abdominal drainage, and PACU length of stay (Table 1).

The nebulization time as well as the residual volume after nebulization was significantly different between groups. The actual dose of ropivacaine nebulized in the peritoneal cavity was 43 ± 7 mg (86%) for the 50 mg group, 93 ± 7 mg (93%) for the 100 mg group, and 115 ± 32 mg (76%) for the 150 mg group, respectively (Table 2).

### 3.1. Primary Endpoint of the Study: Morphine Consumption

We did not find significant differences in the total morphine consumption between groups (Table 3). The majority of patients needed morphine in the PACU (79%) and during the first day after surgery (81%), whereas only half of the patients (44%) used morphine during the second day after surgery. Interestingly, 17% of patients did not require further administration of morphine after PACU discharge.

### 3.2. Secondary Outcome Measures

**Figure 1:**
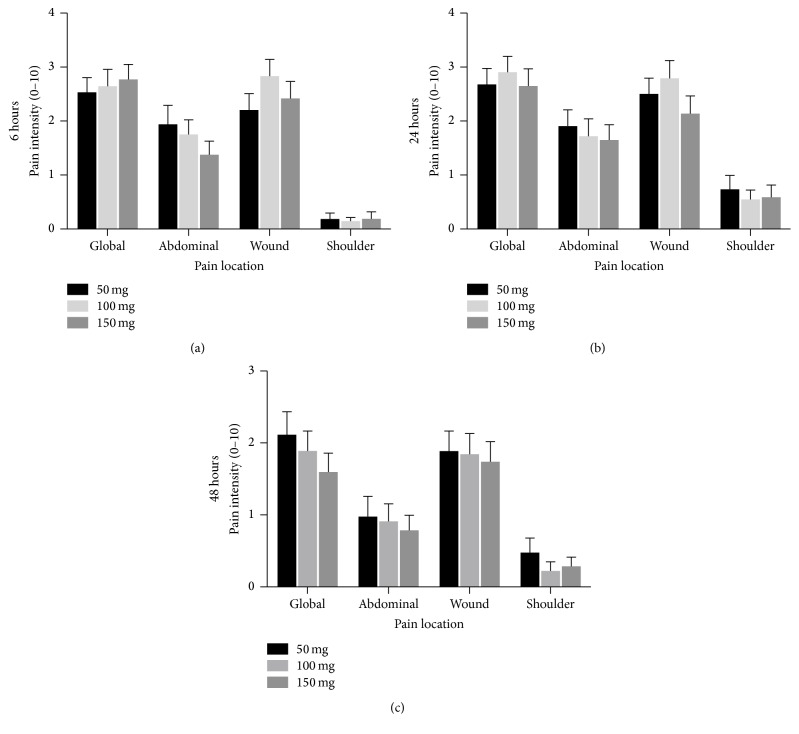
Dynamic pain intensity between the three treated groups 6 hours (a), 24 hours (b), and 48 hours (c) after surgery. Between the three groups, no differences were found in either the intensity of postoperative pain localized at abdominal wall, wound, and shoulder (*P* > 0.05). Data are presented as mean ± standard deviation.

We did not find significant differences between groups in pain intensity after surgery at any evaluation time or place (i.e., abdominal wall, wounds, and shoulder) (Figure 1). The mean pain intensity at the wounds and in the abdomen was similar and higher than the referred pain in the shoulder.

There were no significant differences in the hospital stay, in the readiness for discharge (m-PADDS score ≥ 9), or in the incidence of nausea or vomiting among the three groups (Table 4). There was a strong relationship between the dose of ropivacaine and the incidence of shivering after surgery. One patient from the 50 mg group (2%), four patients from the 100 mg group (8%), and 11 patients from the 150 mg group (21%) shivered in PACU (*P* = 0.004).

### 3.3. Pharmacokinetics Assessment

We were unable to detect ropivacaine in the plasma of seven patients (1 patient in the 50 mg group, 4 patients in the 100 mg group, and 2 patients in the 150 mg group). Mean plasma concentrations of ropivacaine among the three groups were not significantly different, while they are never reaching toxic values (Table 5). Peak plasma concentrations were detected between 20 and 40 minutes after the end of nebulization, followed by a plateau at 60–90 minutes, in all groups. In 5 patients, peak time was detected at 240 or even 360 minutes after the end of nebulization. No clinical signs of toxicity or adverse events were observed.

## 4. Discussion

In this multicenter, double-blind, randomized, controlled, phase III clinical trial, we compared the analgesic effects and the pharmacokinetics of intraperitoneal nebulization of 50 mg, 100 mg, and 150 mg of ropivacaine using the Aeroneb Pro system in patients undergoing laparoscopic cholecystectomy. Increasing the dose of ropivacaine from 50 mg to 150 mg did not reduce the use of morphine after surgery. The absorption of ropivacaine and the pharmacokinetics profile was independent of the dose, and plasma concentrations remained within safe limits in all cases. The increase of the dose of ropivacaine was not associated with a reduction of pain intensity, incidence of PONV, or readiness for discharge, but was associated with the incidence of shivering after surgery.

### 4.1. Morphine Consumption

Peritoneal nebulization of ropivacaine was associated with reduced morphine consumption when compared with both placebo and peritoneal instillation in different clinical scenarios [12–14]. Our data suggest lack of dose-dependent reduction on morphine requirements after surgery. The total consumption of morphine in this study was consistent with the findings in previous studies of ropivacaine nebulization for pain prevention after laparoscopic cholecystectomy. Bucciero et al. reported that patients receiving 60 mg of nebulized ropivacaine consumed 16 ± 12 mg of morphine in the first two days after surgery [19]. In the series of Ingelmo et al., the mean morphine consumption after receiving 30 mg of ropivacaine was 12 ± 15 mg [12, 18]. As in our study, less than half of the patients required morphine during the second day, and a significant proportion of patients did not require morphine after discharge from PACU. Collectively, these results suggest the uselessness of increasing the dose of ropivacaine to reduce the mean morphine consumption after laparoscopic cholecystectomy.

### 4.2. Pain after Surgery

We did not find differences between groups in the pain intensity after surgery. Nebulization of ropivacaine was associated with both statistical and clinical significant reduction of pain intensity after laparoscopic surgery when compared with nebulization of saline after laparoscopic surgery [12–14].

Postoperative pain after laparoscopic cholecystectomy has both a somatic and a visceral origin. Somatic pain is usually well localized to the surgical wounds. Visceral pain is poorly discriminated and involves the entire abdomen or is referred to as the shoulder due to irritation of the diaphragmatic peritoneum. As in previous studies, the nebulization of ropivacaine was able to prevent shoulder pain considerably [16, 18, 19].

Patients in our study received a significant amount of CO_2_ during surgery. Pain after laparoscopic surgery could be influenced by the temperature, humidity, and volume of insufflated [3]. Several devices based on heated or on semipermeable membrane evaporation have been used for humidification and warming of insufflated airway gases. However these devices are unable to efficiently deliver local anesthetic including ropivacaine both in in vitro and in clinical trials [20, 21]. These results could be explained with the physical principle that evaporation enables only evaporation of the solvent (e.g., water) and not of the solute (e.g., local anesthetic). Thus, although these devices are efficient in humidification, they are inappropriate for delivering local anesthetic. By the contrary, the Aeroneb device allows for simultaneous humidification [22].

### 4.3. Nebulization Methods and Doses

The higher the dose of ropivacaine, the higher the residual local anesthetic in the nebulization unit and within insufflating tubes, resulting in hinder parts of the drug from reaching its site of action and to greater wastage of ropivacaine [13, 17, 22]. Nebulization of higher doses of ropivacaine required more time due to the fix nebulization rate of the nebulization system. Nebulization of 50 mg of ropivacaine required less than 15 minutes and had no impact on surgical time as suggested by our and previous results [12, 18, 19].

We used ropivacaine 1% for the study, since it is the most concentrated formulation available in the clinical practice in Italy. The higher the local anesthetic concentration, the lower the nebulization time and consequently the lesser the “fog” in the abdominal cavity. We decided to use 50 mg as the minimum dose for this study because it is an in-between quantity of the doses used in our previous studies [12–14]. This initial dosage, proved to be effective, allowed for double the doses up to what we considered useful in clinical practice.

Our data suggest that increasing the doses of ropivacaine over 50 mg is useless, since it only increases the time of nebulization and the preperitoneal loses of local anesthetics without a proportional increase of the beneficial effects.

### 4.4. Pharmacokinetics Evaluation

We did not identify significant differences on *C*_max_, *T*_max_, and the AUC, among the three administered dosages. The plasma concentrations remained consistent under the mean toxic plasma concentration described in healthy volunteers (4.3 mg/L, range 3.4–5.3 mg/L) [23]. Our data are similar to those of previous animal and human studies [16, 17].

The peak time was reached between 20 and 40 minutes after the end of administration, except for 5 patients in whom we measured a second peak at 240 or 360 minutes. This late peak of concentration could be explained on different local anesthetic absorption during real life surgical conditions. The absorption of ropivacaine could vary depending on the increased peritoneal drug permeability after surgical dissection, reduced intraperitoneal pressure after CO_2_ desufflation with reduction of peritoneal vessels compression, instillation drug amount condensed into connectors, a delayed absorption of ropivacaine collected in the abdominal cavity, and so forth.

When the absorption process is not a limiting factor, the half-life is a hybrid parameter controlled by plasma clearance and distribution volume. If the process is limited, the elimination half-life reflects mainly the rate and extent of absorption and not the elimination process (flip-flop kinetics) [24]. As the mean drug half-life was less than 5 hours, we can posit that the drug had a rapid washout, eliminating the risk of accumulation, interaction with other drugs, or long-lasting cytochromes inhibition.

It is somehow unexpected not to find differences in ropivacaine uptake with a 3-fold increase in the amount of nebulized local anesthetic. This may be due to preperitoneal and postperitoneal losses. In the preperitoneal loss, the local anesthetic never arrived to the peritoneal cavity because it was not nebulized or remained entrapped in the connecting tubes. Since the abdominal cavity is not a close system during surgery, the local anesthetic may be vented out of the abdomen during the surgical procedure. For example, when the surgeon removes the trocars, there is a significant amount of the aerosolized local anesthetic flowing out of the abdomen that will not be absorbed.

We were not able to detect ropivacaine in the plasma of seven patients, as plasma concentrations of ropivacaine were below the limit of quantification (LOQ = 0.1 mcg/mL). Due to the absence of a statistically significant difference regarding the main endpoint of the study among patients in whom plasma concentrations were measured and those in which they were below the limit of quantification, we posit that the local action is greater than the systemic one that, if present, is minimal.

By carefully analyzing the pharmacokinetics and clinical data, our results suggest that the lack of clinical difference in analgesic efficacy, as well as the unexpected absorption profile of ropivacaine, is just part of the current method of nebulization (fix amount per minute) site of effect (most likely sensitive fibers in the peritoneum surface) and absorption. The similar rate of absorption between groups is a further proof on how increasing the dose only leads to increased losses and unchanged delivery of local anesthetic.

### 4.5. Adverse Effects

We did not find major clinically evident side effects during or after surgery. Patients receiving higher doses of ropivacaine (i.e., 150 mg group) presented a significant incidence of shivering in the PACU. Shivering is a common finding during spinal and epidural anesthesia due to vascular dilatation caused by the sympathetic blockade, inducing heat distribution from central to peripheral body areas. We suggest that nebulization realizes a similar mechanism, based on a dose-dependent vascular dilatation [13].

### 4.6. Limitations

A possible limitation of this study is the lack of a control group. The decision not to have a control group was based on previous studies that were held in similar clinical conditions, which had demonstrated that ropivacaine nebulization reduced pain and morphine consumption when compared with placebo [12, 18].

Patients undergoing laparoscopic cholecystectomy remained in the hospital for two days due to institutional policy at the time of the recruiting phase. Therefore, we could not assess the potential benefits of the different doses on the actual discharge time. However, according to a modified postanesthetic discharge scoring system, 40% of patients were ready for hospital discharge 6 hours after surgery and 80% after 24 hours.

Theoretically, a comprehensive pharmacokinetic study should have included intravenous administration of ropivacaine. This point involves important ethical issues that hinder the application of the model in the clinical scenario. Additionally, this is not a pure pharmacokinetic study, but a clinical study with concomitant assessment of ropivacaine exposure. The aim was not a detailed description of ropivacaine kinetics, but to test whether nebulization of ropivacaine at higher doses lead patients to be exposed to toxic concentrations and/or clinical toxicity. We therefore did not attempt to formulate a PK modeling with IV administration, but to analyze systemic exposure and quantify the effect of local anesthetic reuptake on pain and toxicity.

### 4.7. Strengths and Future Directions

We were able to demonstrate the lack of a dose-dependent effect and the futility of higher doses of ropivacaine, along with safety profile of nebulized ropivacaine after laparoscopic cholecystectomy. We were also able to describe the effects of ropivacaine nebulization in the different components of pain after laparoscopic cholecystectomy.

Future technical developments should be oriented to produce devices that enable faster nebulization closer to the umbilical port, ideally without significant fog during the procedure.

## 5. Conclusions

Higher doses of nebulized ropivacaine were not associated with a reduction of morphine consumption or in pain intensity after laparoscopic cholecystectomy. Moreover, the higher the dose of ropivacaine, the higher the time of nebulization, the wither the losses of preperitoneal local anesthetics, and the greater the incidence of shivering after surgery. Intraperitoneal nebulization of ropivacaine was associated with a better control of shoulder pain, than abdominal or wound pain. The plasma concentration of ropivacaine remained within safe margins during and after nebulization with the Aeroneb Pro system.

## Figures and Tables

**Table 1:** Clinical and surgical characteristics. Data are presented as mean ± standard deviation or as percentage (%).

Population	50 mg (*n* = 54)	100 mg (*n* = 52)	150 mg (*n* = 53)
Age (years)	53 ± 13	48 ± 14	52 ± 12
Sex (F/M)	29/25	34/17	34/19
Surgery time (min)	81 ± 28	85 ± 24	74 ± 27
Mean abdominal pressure (mmHg)	13 ± 1	13 ± 1	13 ± 1
CO_2_ total volume (L)	124 ± 72	140 ± 89	126 ± 82
Drain presence	66%	60%	60%
PACU permanence (min)	44 ± 15	43 ± 17	42 ± 17
Body temperature variation (°)	−0.2 ± 0.4	−0.2 ± 0.4	−0.1 ± 0.4

**Table 2:** Nebulization variables. Data are presented as mean ± standard deviation.

	50 mg	100 mg	150 mg	*P* value
	(*n* = 54)	(*n* = 52)	(*n* = 53)	
Nebulization time (min)	16 ± 8	30 ± 18	36 ± 19	<0.001
Residual nebulization volume (mL)	0.2 ± 0.3	0.9 ± 1.2	2 ± 2	<0.001
Effective nebulization volume (mL)	4.8 ± 0.3	9.1 ± 1	13 ± 2	<0.001
Nonadministered ropivacaine (%)	4	9	14	<0.001

**Table 3:** Morphine consumption and proportion of patients receiving morphine during the first two days after surgery. Data are presented as mean ± standard deviation and percentages.

Morphine	50 mg	100 mg	150 mg	*P* value
	(*n* = 54)	(*n* = 52)	(*n* = 53)	
Consumption (mg)				
PACU	4 ± 3	4 ± 4	4 ± 3	0.845
First day	8 ± 9	7 ± 7	7 ± 11	0.845
Second day	3 ± 4	2 ± 5	2 ± 4	0.586
Total	15 ± 12	13 ± 12	13 ± 14	0.756

Proportion of patients receiving morphine (%)				
PACU	80%	75%	83%	0.596
4 h	66%	61%	71%	0.607
6 h	69%	73%	67%	0.825
24 h	83%	84%	81%	0.911
48 h	50%	43%	38%	0.511

**Table 4:** Ambulation and discharge criteria. Data are presented as mean ± standard deviation and percentage.

	50 mg	100 mg	150 mg	*P* value
Patients ambulating at 6 hours (%)	46%	54%	49%	0.712
Time to unassisted walking (hours)	13 ± 9	10 ± 9	11 ± 9	0.338
mPADSS score > 8 at 6 hours	40%	42%	43%	0.947
mPADSS score > 8 at 24 hours	76%	82%	81%	0.682
Ready for discharge (hours)	22 ± 14	19 ± 14	22 ± 7	0.886
Hospital stay (days)	2.4 ± 0.8	2.3 ± 0.7	2.3 ± 0.1	0.910

**Table 5:** Pharmacokinetic data. Data are presented as mean ± standard deviation.

	50 mg	100 mg	150 mg
	(*n* = 12)	(*n* = 12)	(*n* = 13)
*C*_max_ (mcg/mL)	1.8	1.76	2.01
Mean *C*_max_ (mcg/mL)	0.52 ± 0.45	0.59 ± 0.51	0.79 ± 0.66
AUC (mcg/mL*∗*min)	131 ± 152	86 ± 34	115 ± 91
*T* ^1/2^ (min)	281 (72–405)	224 (221–246)	167 (123–354)
Undetected ropivacaine in plasma	1	4	2
